# The proteome of mouse vestibular hair bundles over development

**DOI:** 10.1038/sdata.2015.47

**Published:** 2015-09-15

**Authors:** Jocelyn F. Krey, Nicholas E. Sherman, Erin D Jeffery, Dongseok Choi, Peter G. Barr-Gillespie

**Affiliations:** 1 Oregon Hearing Research Center & Vollum Institute, Oregon Health & Science University, Portland 97239, OR, USA; 2 W.M. Keck Biomedical Mass Spectrometry Lab, University of Virginia, Charlottesville 22908, VA, USA; 3 Department of Public Health & Preventive Medicine, Oregon Health & Science University, Portland 97239, OR, USA

**Keywords:** Proteomics, Hair cell, Transduction

## Abstract

Development of the vertebrate hair bundle is a precisely orchestrated event that culminates in production of a tightly ordered arrangement of actin-rich stereocilia and a single axonemal kinocilium. To understand how the protein composition of the bundle changes during development, we isolated bundles from young (postnatal days P4-P6) and mature (P21-P25) mouse utricles using the twist-off method, then characterized their constituent proteins using liquid-chromatography tandem mass spectrometry with data-dependent acquisition. Using MaxQuant and label-free quantitation, we measured relative abundances of proteins in both bundles and in the whole utricle; comparison of protein abundance between the two fractions allows calculation of enrichment in bundles. These data, which are available via ProteomeXchange with identifier PXD002167, will be useful for examining the proteins present in mammalian vestibular bundles and how their concentrations change over development.

## Background & Summary

The vertebrate hair bundle, the sensory organelle of the inner ear, is necessary for mechanotransduction, which is the process of converting mechanical signals like sound and head movement into electrical excitation in the nervous system. Jutting from the apical surface of a sensory hair cell, the bundle is composed of dozens to hundreds of actin-filled stereocilia and a single true axonemal cilium, the kinocilium^1^. Knowledge of the composition of the bundle is necessary for understanding how the bundle functions and how it is assembled. Mass spectrometry has emerged as the best high-throughput method for characterizing its protein composition. While we have an excellent understanding of the proteome of chick vestibular hair bundles^2^, as well as a more limited view of chick cochlea bundles^3^, determination of the protein composition of mouse hair bundles is an essential next step. Not only is the mouse inner ear an outstanding model in which to study human deafness and balance disorders, but the many genetic and molecular tools available for the mouse make possible many experiments that cannot be carried out in other organisms. Moreover, relatively little is known about how the protein composition of the bundle changes during development; for example, proteins that control key steps in stereocilia elongation may only be found in young bundles.

Hair bundles are scarce; there are only a few thousand hair cells in each auditory or vestibular organ, and in chicken (and mouse; see below), bundles account for less than 1% of the total protein in the organ^2^. Only ~2 ng of actin is present in the bundles of one mouse utricle^4^, and actin accounts for >50% of the total bundle protein (see below). Moreover, many of the most interesting molecules in the stereocilia are present at a molar abundance <10^−5^ that of actin—less than one attomole per ear. Thus biochemical experiments to characterize the bundle's proteome require extensive dissection, specialized purification methods, and use of sensitive detection methodologies.

Protein mass spectrometry has emerged as the only technique with the sensitivity and dynamic range to analyse a broad range of proteins from hair bundles. While top-down^5^ and data-independent^6^ methods for protein analysis are likely to develop sufficiently for analysis of bundle proteins in the future, at present data-dependent shotgun mass spectrometry^7^ offers a well-characterized approach that allows detection of both low- and high-abundance proteins, albeit ultimately limited by the dynamic range of mass spectrometers. In addition, methods for quantifying proteins using mass spectrometry data have improved, allowing accurate measurement of relative protein abundance using either parent- or daughter-ion intensities^8,9^. Together these techniques allow thorough characterization of the proteome of the hair bundle.

**Figure 1:**
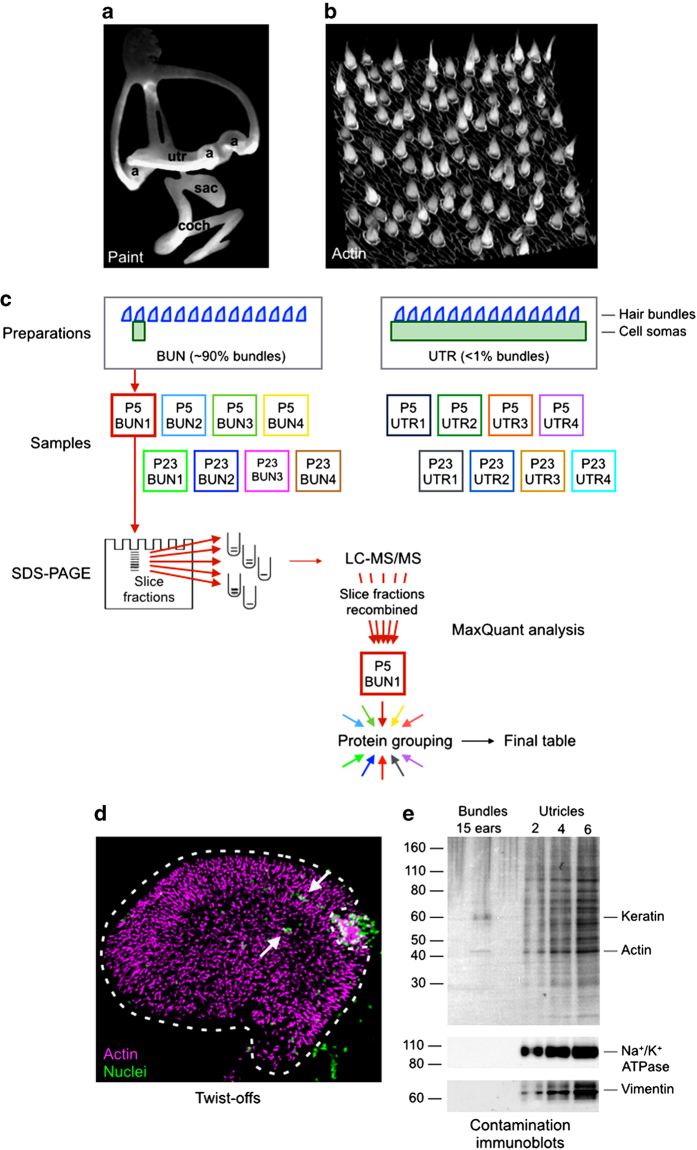
Sample and data processing workflow for mass spectrometry analysis of purified mouse utricle hair bundles and utricles. (**a**) Paint-fill of the mouse inner ear, with the sensory patches marked (‘utr’, utricle; ‘a’, ampullae of the semicircular canals; ‘sac’, saccule; ‘coch’, cochlea). Adapted from Fig. 1b of ref. 22 with permission. (**b**) Phalloidin stain (to detect actin) of a ~50×50 μm region of the utricular epithelium (~100 hair cells, out of ~3,000). Hair bundles are seen rising out of the utricular sensory epithelium, which is marked by circumferential actin belts surrounding each cell. (**c**) Workflow. Four biological replicates each of P5 bundles (BUN), P23 bundles, P5 utricles (UTR), and P23 utricles were prepared. Of the bundle samples,~10% of the protein derived from the utricle; bundles make up <1% of the protein in the utricle sample. Proteins of each of the sixteen samples were separated using short SDS-PAGE runs, and the lanes were cut into six pieces each. Samples were reduced, alkylated, and subjected to trypsin digestion prior to LC-MS/MS. A total of 96 LC-MS/MS runs were carried out in the project. Peptides from each run were identified using MaxQuant; data from the six gel slices per protein were combined back together so the final output represented all peptides detected from the single biological replicate. MaxQuant grouped all proteins that were not distinguished by unique peptides; our additional grouping brought together all proteins that shared at least 20% of their peptides. (**d**) Purified mouse utricle hair bundles stained with phalloidin to detect actin (magenta) and DAPI to detect nuclei (green). Both bundles and areas of substantial cellular contamination can be seen in the dissecting microscope, and contaminating regions can be cut away from bundles. For the preparation shown, the area that would be excised is indicated by dashed lines. Note that some nuclear contamination would remain (arrows). (**e**) Protein immunoblot used to estimate contamination in mouse bundle preparations. The indicated number of ear-equivalents of bundles (left) or utricular epithelia (right) were run. The top panel is the India ink stain of the blot after protein transfer; note the bands corresponding to actin and contaminating keratin in the bundles lane. The bottom two panels are protein immunoblots. Even with 15 ears' worth of material, the plasma-membrane marker Na^+^/K^+^-ATPase and the intermediate-filament protein vimentin were not detected in the bundle preparation.

We used the twist-off hair-bundle purification technique^10^, which captures bundles in a matrix of agarose gel, to isolate mouse bundles from utricles, one of the vestibular organs (Fig. 1a,b). Four biological replicates of bundles, each from 100 ears, were collected from immature (~P5) and young adult (~P23) mice. Purified bundle proteins were subjected to liquid chromatography-tandem mass spectrometry (LC-MS/MS) using an Orbitrap mass spectrometer. To provide a reference for examining bundle enrichment, we also analysed four biological replicates of 10 mouse utricles at each of the same two developmental stages. We identified proteins with the Andromeda search engine and quantified them using label-free measurements of the precursor peptide intensity. By comparing the relative abundance of each protein identified in both bundles and utricle, we were able to determine the protein's enrichment in hair bundles.

## Methods

### Experimental design

We collected four sets of samples, which included purified mouse hair bundles from postnatal day 4–6 mice (referred to as ‘P5’) and P21-P25 utricles (‘P23’), as well as utricles from the same ages (Table 1). Bundle samples are referred to as ‘BUN’; they are modestly contaminated by cell bodies. Utricle samples are referred to as ‘UTR’; they also contain hair bundles, albeit at <1% of the total protein (Supplementary Fig. 1). Four biological replicates were prepared for each of the four conditions. Proteins were partially separated by SDS-PAGE, then digested into peptides. LC-MS/MS was used to identify and quantify peptides from the four conditions. We used MaxQuant^8^, with its built-in Andromeda search engine^11^, to match peptides to mouse protein database entries, to assemble peptides into proteins, and to quantify those proteins. The workflow for bundle isolation, sample preparation, mass spectrometry, and data analysis is shown in Fig. 1c. Information about the mass spectrometer instrument settings, database searching, peptide-to-protein mapping, and quantitation (satisfying Minimal Information About a Proteomics Experiment [MIAPE] requirements^12^) is presented in Supplementary Table 1.

### Sample preparation

Hair bundles were purified using the twist-off method^2,4,10,13,14^; methods are described in detail in ref. 14. Utricle maculae were dissected from CD-1 mice at P4-P6 (developing utricle) or P21-P25 (young adult). Methods for dissecting mouse utricles have been presented elsewhere^15,16^. Maculae were adhered to the untreated bottom of a 35 mm plastic dish (EASY GRIP Falcon Petri dishes; Becton Dickinson) in Leibovitz's L-15 Medium without phenol red (21083-027; Thermo Life Technologies). Otolithic membranes were removed with an eyelash. A plastic washer was placed around the maculae on the dish, and the preparation was flooded with 4.5% low-melting point agarose (filtered through a 5 μm filter) at 42 °C. The agarose was allowed to set to a firm gel at 4 °C, then the disk of agarose formed by the ring was placed on a perfusion platform under a dissecting microscope. Under constant flow with L15, the maculae were removed, leaving hair bundles stuck in the agarose. A small scalpel or tungsten needle was used to clear away obvious cellular debris (Fig. 1b), then bundles from a single utricle were removed in an agarose plug of minimal volume (<0.5 μl). Isolated bundles in agarose were frozen at −80 °C in low-protein-binding microfuge tubes. Bundle samples were later pooled for mass spectrometry analysis.

For analysis of whole utricular maculae, the utricle was dissected to remove all tissue outside the sensory epithelium region and the otolithic membrane was removed. Unlike in our previous analyses of chick utricles^2,17^, where we peeled the sensory epithelium off of the basement membrane and connective tissue, we analysed whole unpeeled mouse utricles here. Mouse utricle sensory epithelium does not readily peel off the stroma without a protease treatment (e.g., collagenase or thermolysin). In addition to hair cell and supporting cell proteins, the preparation thus contained proteins derived from extracellular matrix, stroma, and nerve.

### Hair-bundle characterization

To examine isolated hair bundles by fluorescence microscopy, the agarose disk containing cleaned isolated hair bundles was washed in L15 medium in a 12-well plate. The agarose disk was then transferred to 4% formaldehyde in PBS and incubated for 20 min at room temperature. After washing 2x with PBS, disks were stored at 4 °C overnight. The next day, the bundles were permeabilized with 0.5% Triton X-100 in PBS for 10 min and then transferred to 13 nM (1:500 of 6.6 μM stock) Alexa Fluor 488 Phalloidin (Life Technologies) and 1:10,000 DAPI (Sigma) in PBS for 2–3 h. Disks were rinsed 3x in PBS, then bundles in agarose were removed from the disk in a thin slab. After placing on a slide, the sample was coated with VECTASHIELD mounting medium (Vector Laboratories) and covered with coverslip.

For immunoblotting characterization, mouse hair bundles and utricles were collected from P21–25 mice as described for the mass spectrometry experiments. Bundle and utricle proteins were solubilized with reducing NuPAGE LDS sample buffer (Invitrogen); the samples were heated to 65 °C for 15 min, then 95 °C for 5 min, and were separated by running into a NuPAGE 4–12% Bis-Tris gel (1.5 mm×10 well; Invitrogen). Proteins were transferred onto a PVDF membrane (Millipore) following standard procedures. The membrane was rinsed with water and PBS/0.1% Tween-20 (PBST) and then stained for 30 min with Black Indian ink (Windsor & Newton) diluted 1:5,000 in PBST. The membrane was then blocked for 1 h in Amersham ECL prime blocking reagent (GE Healthcare) and incubated overnight at 4 °C with primary antibodies from Developmental Studies Hybridoma Bank (anti-vimentin, #AMF-17b; ATPase, anti-Na(+) K(+) alpha subunit, #a5) diluted 1:1,000 in blocking reagent. After 5×6 min washes in PBST, the membrane was incubated for 1 h at room temperature in secondary antibodies (goat anti-rabbit HRP and goat anti-mouse HRP) diluted 1:10,000 in blocking reagent. Bands were visualized using SuperSignal Pico West ECL reagent (Thermo Scientific).

### Sample preparation for mass spectrometry

To remove interfering polymers from the agarose used for bundle isolation, we separated proteins by a short SDS-PAGE run prior to reduction, alkylation, and trypsin digestion^2,18^. Utricle proteins were also separated by SDS-PAGE. To prepare samples, 1.2x NuPAGE LDS sample buffer (Invitrogen) with 50 mM dithiothreitol was added to a combined final volume of 40–60 μl per 100 utricles' worth of bundles; the volume varied from sample to sample depending on how much agarose the bundles were excised with. Utricle proteins were also solubilized with NuPAGE LDS sample buffer (40 μl per 10 utricles). The samples were heated to 65 °C for 15 min, then 95 °C for 5 min. Proteins were separated by running ~1 cm into a NuPAGE 4–12% Bis-Tris gel (1.5 mm×10 well; Invitrogen); one lane was used for samples of <50 μl and two lanes were used for samples of >50 μl. Gels were rinsed with water, then stained for 5 h with Imperial Protein Stain at room temperature (Thermo Scientific). After rinsing with water at 4 °C over 5 h, 1 cm of gel with separated proteins was manually sliced into six pieces, each of which was processed separately in the subsequent steps.

Gel pieces were transferred to siliconized tubes, then were washed with 200 μl HPLC-grade water (vortexed 30 s), 200 μl of 50% 50 mM NH4HCO3/50% MeOH (vortexed 1 min), 200 μl of 50% 50 mM NH_4_HCO_3_/50% acetonitrile (vortexed 5 min), and 200 μl of 100% acetonitrile (vortexed 30 s). The remaining solution was removed and the gel pieces were dried briefly in a SpeedVac vacuum concentrator; they were stored overnight at −20 °C.

The gel pieces were rehydrated with 100 μl of freshly made 25 mM DTT in 50 mM NH_4_HCO_3_, then were incubated for 20 min at 56 °C. The supernatant was discarded and 100 μl of 55 mM iodoacetamide in 50 mM NH_4_HCO_3_ was added; this solution was incubated with the gel pieces in the dark for 20 min at RT. The supernatant was discarded and the gel pieces were washed twice with 400 μl HPLC-grade water. The washes were discarded and 200 μl 50% 50 mM NH_4_HCO_3_/50% acetonitrile was added (vortexed 5 min). This wash was discarded and 200 μl 100% acetonitrile was added (vortexed 1 min). The supernatant was discarded and samples were dried in the SpeedVac for 2–3 min.

We found that digestion in ProteaseMAX Surfactant-Trypsin Enhancer (Promega) substantially increased the recovery of hair-bundle peptides. A 1% solution of ProteaseMAX was prepared by adding 100 μl of 50 mM NH_4_HCO_3_ to the stock aliquot with swirling to mix; this ProteaseMAX solution was kept on ice. A 1.5 ml aliquot of 0.01% ProteaseMAX was prepared by adding 15 μl 1% ProteaseMAX to 1485 μl 50 mM NH_4_HCO_3_. A 1.5 ml aliquot of 0.01% ProteaseMAX/6 ng μl^−1^ trypsin was made by adding 15 μl 1% ProteaseMAX to 1440 μl of 50 mM NH_4_HCO_3_; the solution was mixed, then 45 μl of a 200 ng μl^−1^ trypsin stock (Sigma-Aldrich T6567 proteomics grade, from porcine pancreas, dimethylated), diluted fresh in 50 mM NH_4_HCO_3_, was added. To each gel piece, 30 μl of the 0.01% ProteaseMAX/6 ng μl^−1^ trypsin solution was added and incubated for 30 min at 4 °C. Gel pieces were overlaid with 20 μl of the 0.01% ProteaseMAX solution to maintain them fully submerged. Trypsin digestion was allowed to proceed for 3 h at 37 °C.

The digest solution was transferred to new tubes (25–40 μl of liquid from each tube), and samples were combined if original gel pieces had been split; 30 μl of 2.5% trifluoroacetic acid (in HPLC-grade water) was added to gel pieces (vortexed 15 min). The solution was removed and combined with the initial digest solution. The solution was vortexed and then the combined solution was centrifuged for 10 min at 14,000 rpm in a microcentrifuge. The supernatant was transferred to 0.45 μm filter tubes (Millipore Ultrafree centrifugal filters, #UFC0HV00); samples were spun 5 min at 4,000 r.p.m. All samples were dried in the SpeedVac until virtually all solution was evaporated (~2 h), then were stored at −80 °C prior to mass spectrometry.

### Mass spectrometry

A single experiment's worth of hair bundles or utricle was analysed using six LC-MS/MS runs, corresponding to the six gel pieces. The LC-MS/MS system consisted of a Thermo Electron Orbitrap Velos ETD mass spectrometer and a Protana nanospray ion source, which was interfaced to a reversed-phase capillary column of 8 cm length×75 μm internal diameter, self-packed with Phenomenex C18 Jupiter of 10 μm particle size. Samples were brought up with 15 μl of 50% acetonitrile/5% formic acid; 7.5 μl was injected and peptides eluted from the column by an acetonitrile/0.1 M acetic acid gradient at a flow rate of 0.5 μl min^−1^ over 1.2 h. The nanospray ion source was operated at 2.5 kV. The digest was analysed using the data-dependent capability of the instrument, acquiring in sequential scans (1) a single full scan mass spectrum in the Orbitrap detector at 60,000 resolution to determine peptide *m/z* and intensities, and (2) 20 product-ion spectra in the ion trap, which allow us to match peptides to the database.

### Mass spectrometry data processing by MaxQuant

MaxQuant version 1.5.1.2 software was used for protein identification and quantitation^8^. The default contaminants file associated with the MaxQuant download was edited to remove entries known to be present in hair bundles (e.g., actin) and to add additional impurities that entered the bundle-purification workflow (e.g., keratins, haemoglobins). Mass spectrometry data were searched against Ensembl version GRCm38_71 (released April, 2013) using Andromeda^11^; the Ensembl FASTA file was supplemented with recently determined *Xirp2* alternative splice products^19^. ‘Match between runs’ was not used. Protein identifications were reported with an false discovery rate (FDR) of 1%.

We used MaxQuant to calculate iBAQ, a measure of protein abundance. The iBAQ value is obtained by dividing protein intensities by the number of theoretically observable tryptic peptides between 6 and 30 amino acids^20^, and is on average highly correlated with protein abundance^9,20^.

### Additional mass spectrometry data processing

We wrote a Mathematica version 10 program to further process the MaxQuant ‘proteinGroups.txt’ file. This program (1) replaced default protein names and symbols with user-defined entries, (2) deleted all entries marked by MaxQuant as a ‘Potential contaminant’ or ‘Reverse’ (proteins labelled as ‘Only identified by site’ were retained, (3) grouped together proteins that share >20% of their peptides, and (4) prepared an output file.

The output file was imported into Excel, where we (5) calculated relative iBAQ (riBAQ)^2,9^, which is the iBAQ for a protein or protein group (calculated by MaxQuant) divided by all non-contaminant, non-reversed iBAQ values for a replicate, (6) determined means and standard deviations for each experimental condition, (7) determined bundle-to-utricle ratios were calculated for each developmental age, and (8) calculated P5-to-P23 ratios for bundles and for utricles.

### Code availability

The custom Mathematica code that takes the MaxQuant output and groups related proteins is available in the ProteomeXchange dataset (PXD002167).

## Data Records

All data and analysis files, described below, have been deposited to the ProteomeXchange repository (accession number PXD002167; http://www.proteomexchange.org). This dataset includes 96. RAW files, representing LC-MS/MS data from all four experimental conditions (P5 bundles, P5 utricle, P23 bundles, P23 utricle) represented by four biological replicates analysed in six runs, one for each gel slice (Data Citation 1). The dataset also includes ‘SEARCH_MAXQUANT.zip’, which contains all of the MaxQuant files from the ‘txt’ folder, as well as the ‘experimentalDesignTemplate.txt’ file that specifies how the files are to be searched by MaxQuant (Data Citation 1). The FASTA file used for the MaxQuant search is included as ‘OTHER_FASTA.zip’ (Data Citation 1), and the Mathematica programs, input file, and outputs are located together as ‘OTHER_MATHEMATICA.zip’ (Data Citation 1). Finally, the most important file for readers not interested in reanalysing the data is ‘S1 Mouse P4-P21 data Orbitrap-MaxQuant 2015-05-29c.xls’, compressed in the data record as ‘OTHER_EXCEL_FINAL.zip’ (Data Citation 1); this is the Excel file that contains the averaged data for each protein under each experimental condition. This table is replicated here as Supplementary Table 2.

## Technical Validation

### Contamination

The utility of the hair-bundle dataset depends on the ability to disregard proteins present in the bundle samples that arose from contamination from cell bodies; dissections are rarely perfect. Some damaged tissue can be removed following examination of each preparation using a dissecting microscope and dark field illumination; we can then dissect away the most egregious and obvious contamination (Fig. 1d). Nevertheless, the isolated bundle samples still have significant levels of proteins from structures that are known not be present in hair bundles, e.g., nuclei and mitochondria.

We assess the purity of the hair-bundle preparation by determining the relative abundance of histones in both bundles and utricle (Table 2); as nuclear proteins should be absent from bundles. The ratio of BUN/UTR for histones represents the fraction of protein present in BUN that originally derived from utricles. Using histone entries that were detected in 4/4 BUN runs and 4/4 UTR runs, we determined that the P5 BUN sample was ~97% bundles, and that the P23 BUN sample was ~84% bundles. The enrichment values for the two ages (0.033±0.027 and 0.16±0.10) can be used to test statistically for significant enrichment of proteins in bundles over these background levels.

In addition, we also assessed purity by carrying out immunoblotting of hair-bundle samples with antibodies against proteins known to be absent from bundles (Fig. 1e). Using P23 samples, bundle signals for Na+/K+-ATPase and vimentin were below the limit of detection in these assays. However, these were separate preparations than those analysed by mass spectrometry; the histone analysis of the P5 and P23 mass spectrometry data are more relevant for assessing contamination in those preparations.

### Comparison of biological replicates for each condition

**Figure 2:**
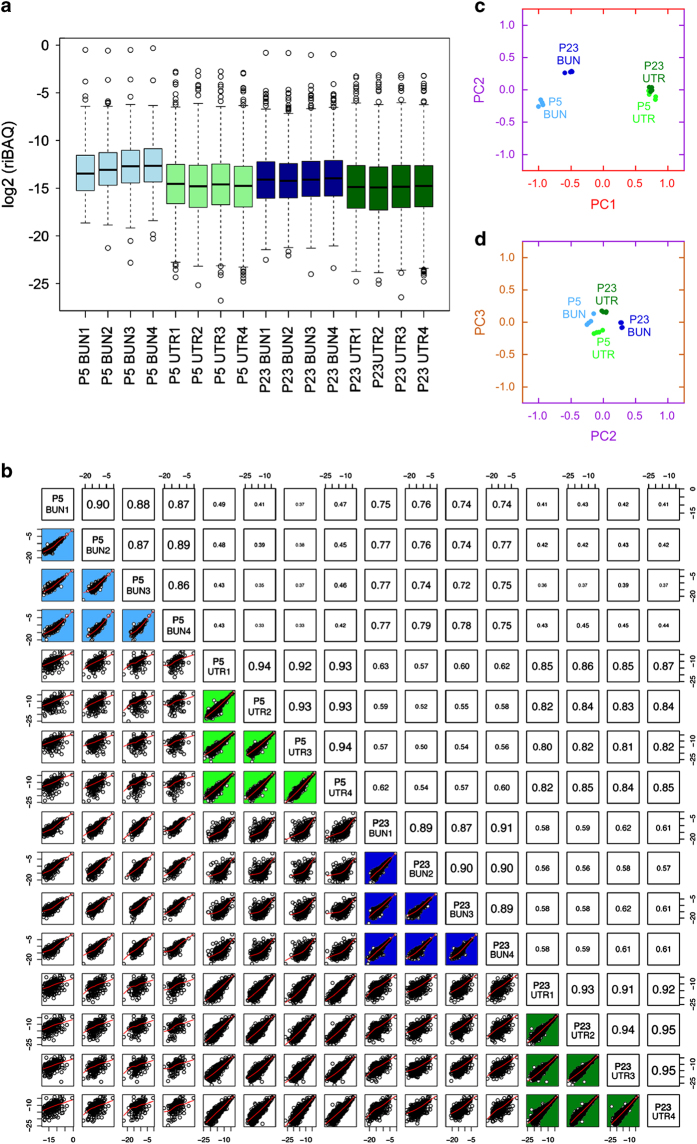
Comparison of the sixteen samples. (**a**) Box plots showing riBAQ values for all proteins in the indicated samples. (**b**) Matrix scatter plot showing pairwise comparisons of proteins detected in each of two samples. Red lines, LOESS smoothing of data. Numbers on the right/upper side of the diagonal are the absolute values of the Pearson’s correlation coefficients. Note the higher correlations for biological replicates (coloured background). (**c**,**d**) Principal component analysis of all 16 samples. In (**c**), PC1 (91% of variance) is plotted against PC2 (5% of variance). In (**d**), PC2 is plotted against PC3 (2% of variance).

Four biological replicates were used for the four conditions (P5 BUN and UTR, P23 BUN and UTR). Box plots showing the distribution of riBAQ values for each of the 16 samples are shown in Fig. 2a. A matrix scatter plot with pairwise comparisons of all samples is shown in Fig. 2b. Note that the biological replicate samples showed highest Pearson correlation coefficients (numbers in boxes). Similarly, principal component analysis showed that each of the four sets of samples were clearly distinguished from each other (Fig. 2c,d). Over 90% of the variance was accounted for by PC1. Bundle samples showed the greatest separation in PC2, and utricle samples in PC3. Together the results in Fig. 2 show that the replicates for each experimental condition were sufficiently similar that they could be combined.

### Distribution of proteins

**Figure 3:**
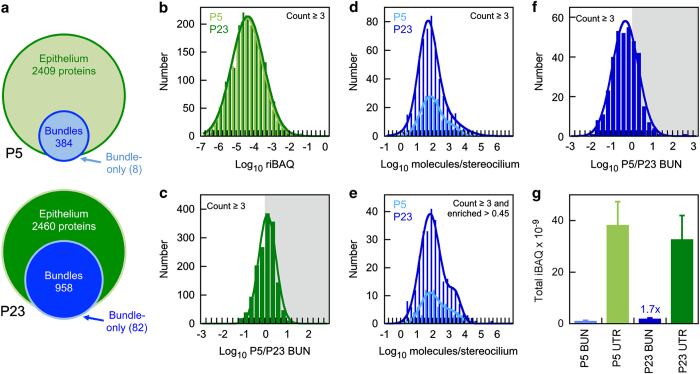
Protein expression patterns. (**a**) Venn diagram showing numbers of proteins or protein groups identified in bundles and utricle samples. A small number of proteins were only identified in bundle. (**b**) Distribution of log_10_ of average protein riBAQ values for utricle samples. Only proteins detected from three or more samples were included. (**c**) Distribution of riBAQ values for each utricle protein at P5 and P23. Fit is a single Gaussian (peak at +0.2). (**d**) Distribution of log_10_ of average protein molecules per stereocilium values for bundle samples. riBAQ values were converted into molecules per stereocilium assuming that riBAQ accurately measured protein fractional abundance and that actin was present at 400,000 molecules per stereocilium^2,9^. Only proteins detected from three or more samples were included. (**e**) Same as (**d**) except only proteins with BUN/UTR enrichment of 0.45 or greater were included. (**f**) Distribution of riBAQ values for each bundle protein at P5 and P23. Fit is a single Gaussian (peak at −0.6). (**g**) Total iBAQ values as proxy for total protein. Contaminants and reversed entries were removed first. P5 and P23 utricle samples were similar, but there was 1.7-fold more iBAQ signal in the P23 bundle sample as compared to P5.

The numbers of proteins identified in bundles and epithelium at each age are indicated in Fig. 3a. The distribution of riBAQ values for utricle proteins was very similar at P5 as compared to P23 (Fig. 3b). The distribution of log P5/P23 ratios for each protein was centred around 0, showing that the majority of proteins changed their expression levels little between P5 and P23 (Fig. 3c). Reflecting the smaller numbers of proteins identified, the distribution of bundle protein riBAQ proteins had a reduced amplitude, and appeared to be a shift towards more abundant proteins too (Fig. 3d), which was most evident when only proteins enriched in bundles at the 0.45 or greater level were plotted (Fig. 3e). Most proteins detected in bundles were more abundant at P23 than at P5 (Fig. 3f). Together these data suggest that a smaller number of abundant proteins dominated the bundle sample at P5, and that during development the bundle proteome became more complex.

As noted above, while similar numbers of proteins were identified in P5 and P23 utricles, less than half as many proteins were identified in P5 bundles as in P23 bundles (Fig. 3a). There are fewer utricle hair cells at P5 than at P23 (ref. 21), which was also reflected in the total iBAQ signal (excluding contaminants and reversed matches); there was nearly two-fold more iBAQ signal in bundles at P23 than at P5 (Fig. 3g).

## Usage Notes

### Data processing with MaxQuant

MaxQuant's built-in Andromeda search engine should be used to match MS2 spectra to database peptides, and proteins will be quantified based on their MS1 intensity profile. Default settings should be used for MaxQuant analysis of the.RAW files with the exception that the iBAQ quantitation option should be checked.

### Data processing with custom Mathematica program

We wrote a Mathematica (version 10.1.0.0) program to process the ‘proteinGroups.txt’ MaxQuant output file further. Two versions of the program are provided; ‘MaxQuant 1.2.2.5 mouse 2013-12-19.nb’ is the version used to analyse the data in this project, while ‘MaxQuant 1.4.1.2 2015-02-25.nb’ was redesigned for more recent versions of MaxQuant and is more robust. This program carries out the following steps:

(1) Replace protein names and symbols. A user file with protein descriptions and symbols associated with each identifier (‘Mouse abbrv table 2013-11-21.txt’) was used to replace those from information provided from the FASTA file. For protein symbols, we use the gene name, all caps and not italic, in all cases. Ambiguous entries were resolved using the ortholog search function in Ensembl and with GeneCards (http://www.genecards.org).

(2) Delete reversed and contaminant entries. All entries marked by MaxQuant as a ‘Potential contaminant’ or ‘Reverse’ were deleted. Proteins labelled as ‘Only identified by site’ were retained.

(3) Group together proteins that share >20% of their peptides. If a set of peptides for a protein was identical to or completely contained within that of another protein, MaxQuant groups those proteins together (‘redundant groups’); the entry with the largest number of peptides was used to identify the redundant group. Redundant groups that shared more than 20% of their identified peptides were further grouped in our analysis (‘shared-peptide groups’); the entry with the greatest intensity associated with it was used to identify the shared-peptide group. These groups are listed in the file labelled ‘_ 5_GROUPS _LIST.txt’.

(4) Prepare output. A new file (‘proteinGroups 2013-12-18c_4_FINAL.txt’) was generated that contains a more limited set of information.

### Data processing with excel

The output from the Mathematica file was imported into Excel. The file deposited at ProteomeXchange has all of the formulas used for processing still intact. The following steps were carried out:

(5) Calculate riBAQ. To generate a relative abundance for each protein in its sample, we divided a protein's iBAQ value by the sum of all non-contaminant iBAQ values for that sample, yielding a relative iBAQ (riBAQ). We have shown that on average, riBAQ is an accurate measure of protein abundance^9^.

(6) Calculate mean and standard deviation for biological replicates. We calculated mean and standard deviations on riBAQ values, not on their logarithmic transformations.

(7) Calculate BUN/UTR enrichment at each developmental age. These enrichment values allow for sorting of proteins that are likely to be true bundle components from those that are contaminants.

(8) Calculate P5/P23 enrichment. These ratios allow identification of developmentally regulated proteins.

## Additional Information

**How to cite this article:** Krey, J. F. *et al.* The proteome of mouse vestibular hair bundles over development. *Sci. Data* 2:150047 doi: 10.1038/sdata.2015.47 (2015).

## Supplementary Material



Supplementary Figure 1

Supplementary Table 1

Supplementary Table 2

## Figures and Tables

**Table 1:** Samples analysed for mass spectrometry.

**Sample name**	**Developmental age range**	**Tissue source**	**Biological replicates**	**Gel slices per replicate**
P5 BUN	P4-P6	Mouse utricle hair bundles	4	6
P5 UTR	P4-P6	Mouse utricles	4	6
P23 BUN	P21-P25	Mouse utricle hair bundles	4	6
P23 UTR	P21-P25	Mouse utricles	4	6

**Table 2:** Histone contamination of hair-bundle samples

**Identifier**	**Best protein description**	**Best protein symbol and all other associated symbols**	**P5 BUN/ UTR**	**P23 BUN/ UTR**
ENSMUSP00000045816_family	Family (Histone cluster 1, H1c)	HIST1H1C; HIST1H1A; HIST1H1D; HIST1H1E; HIST1H1T	0.006	0.021
ENSMUSP00000125754_family	Family (H3 histone, family 3A)	H3F3A; H3F3B; HIST1H3A; HIST1H3B; HIST1H3C; HIST1H3D; HIST1H3E; HIST1H3F; HIST1H3G; HIST1H3H; HIST1H3I; HIST2H3B; HIST2H3C1; HIST2H3C2	0.069	0.224
ENSMUSP00000136357	Histone cluster 4, H4	HIST4H4	0.043	0.257
ENSMUSP00000106095_family	Family (Histone cluster 1, H2bq)	HIST1H2BQ; HIST1H2BA; HIST1H2BB; HIST1H2BC; HIST1H2BE; HIST1H2BF; HIST1H2BG; HIST1H2BH; HIST1H2BJ; HIST1H2BK; HIST1H2BL; HIST1H2BM; HIST1H2BN; HIST1H2BP; HIST1H2BR; HIST2H2BB; HIST2H2BE; HIST3H2BA	0.040	0.199
ENSMUSP00000089336_family	Family (Histone cluster 1, H2ah)	HIST1H2AH; H2AFJ; H2AFX; HIST1H2AA; HIST1H2AB; HIST1H2AC; HIST1H2AD; HIST1H2AE; HIST1H2AF; HIST1H2AG; HIST1H2AI; HIST1H2AK; HIST1H2AL; HIST1H2AN; HIST1H2AO; HIST1H2AP; HIST2H2AA1; HIST2H2AA2; HIST2H2AC; HIST3H2A	0.008	0.080
EENSMUSP00000038221	H2A histone family member Y	H2AFY	UTR only	0.030
			*0.037±0.026*	*0.16±0.11*
Only histones or histone groups detected in all 8 bundle and utricle samples at each developmental age were used.				
