# Supplementary figures and images for: The proteome of mouse vestibular hair bundles over development
Source: Sci Data. 2015 Sep 15;2:150047. doi: 10.1038/sdata.2015.47 (PMC4570149; doi:10.1038/sdata.2015.47)

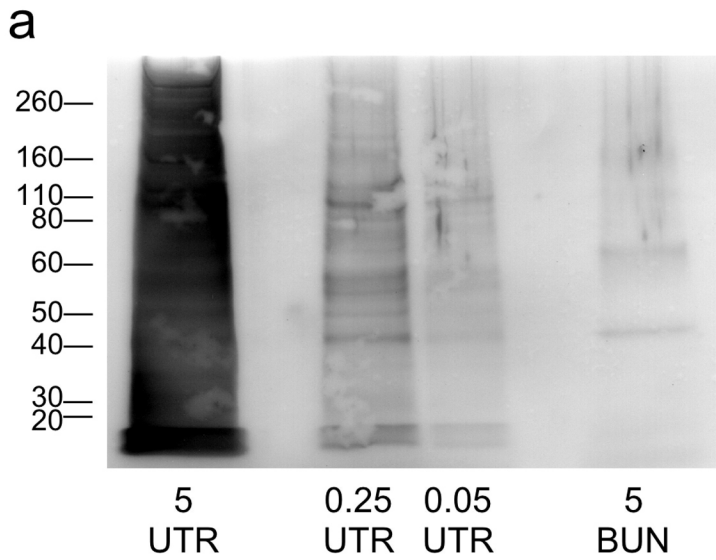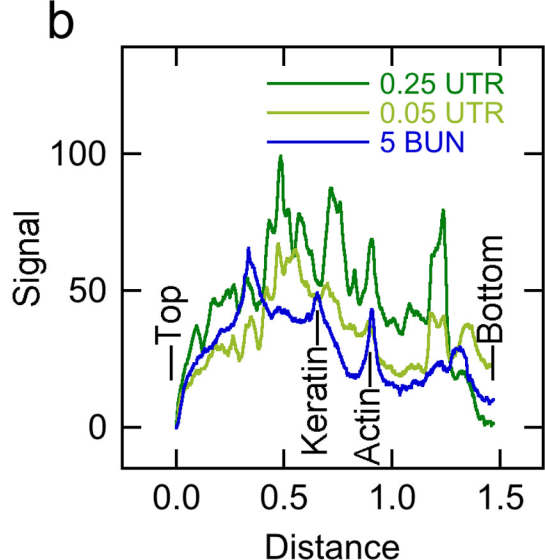

Supplement: Supplementary Figure 1 [file sdata201547-s2.pdf]
