# Supplementary material for: The proteome of mouse vestibular hair bundles over development
Source: Sci Data. 2015 Sep 15;2:150047. doi: 10.1038/sdata.2015.47 (PMC4570149; doi:10.1038/sdata.2015.47)
Supplement: Supplementary Table 2 [file sdata201547-s4.docx]

# MIAPE

# Supplemental information for Krey et al. ("The proteome of mouse vestibular hair bundles over development")

# Mass spectrometry

1. General features

1.1 Global descriptors

– Responsible person (or institutional role if more appropriate); provide name, affiliation and stable contact information: **Dr. Nicholas Sherman, UVA WM Keck Biomedical Research Facility, Jordan Hall Room 1105, 434-924-0070**

– Instrument manufacturer and model :**ThermoFisher Orbitrap Velos ETD**

– Customizations (summary): **none**

2. Ion sources

*As each spectrum is acquired using only one ionization source, select the one that applies*

2.1 Electrospray Ionization (ESI)

– Supply type (static, or fed): **fed**

– Interface manufacturer, model: **Proxeon Nano ES**

– Sprayer type, manufacturer, model: **in-house fused silica with laser-pulled spray tip**

– Other parameters if discriminant for the experiment: **360x75um fused silica packed with 7.5cm of 10um Jupiter C18 packing material**

3. Post-source component

*As an MS spectrum or chromatogram performed on one instrument cannot be acquired using all existing analyzers and detectors, select the elements that apply*

3.1 Analyzer

– Ion optics, ‘simple’ quadrupole, hexapole, Paul trap, linear trap, magnetic sector, FT-ICR, Orbitrap: name of the analyzer(s): **IT, Orbitrap**

3.2 Activation / dissociation

*The associated acquisition parameters are covered in 4.1*

– Instrument component where the activation / dissociation occurs: **IT**

– Gas type (when used) : **Helium**

– Activation / dissociation type : **CID**

4. Spectrum and peak list generation and annotation

4.1 Data acquisition

– Software name and version: **Xcalibur version 1.0.2.65 sp2**

– Acquisition parameters:

**Global Data Dependent Settings:**

**Predict ion injection time enabled**

**Dynamic exclusion enabled**

**Repeat Count: 1 scan**

**Repeat Duration: 30.00 seconds**

**Exclusion List Size: 200 parent ions**

**Exclusion Duration: 60.00 seconds**

**Exclusion mass width by mass**

**Exclusion mass width low: 1.00000 amu**

**Exclusion mass width high: 2.00000 amu**

**Expiration: disabled**

**Scan Event Details:**

**1: FTMS + p res=60000 (300.0-1600.0 m/z)**

**CV = 0.0V**

**2: ITMS + c Dep MS/MS 1^st^ (Nth) Most intense ion from (1)**

**Activation Type: CID**

**Min. Signal Required: 1000.0 ion counts**

**Isolation Width: 3.00 amu**

**Normalized Coll. Energy: 35.0 V**

**Default Charge State: 3**

**Activation Q: 0.250**

**Activation Time: 10.000 ms**

**CV = 0.0V**

**3: repeat step 2 for 2^nd^ through 20^th^ scans (N=2-20)**

**Data Dependent Settings:**

**Parent Mass List: (none)**

**Reject Mass List: m/z 371.00000 445.10000**

**Neutral Loss Mass List: (none)**

**Product Mass List: (none)**

**Neutral loss in top: 3**

**Product in top: 3**

– Chromatography parameters:

**LC gradient: Peptides were eluted from the column by an acetonitrile/0.1 M acetic acid (solvent B) gradient at a flow rate of 0.5 µL/min over 1.25 hours as follows:**

**0-5 min, 2-3% B**

**5-15 min, 3-5% B**

**15-20 min, 5-8% B**

**20-30 min,8-12% B**

**30-40 min,12-16% B**

**40-50 min, 16-21% B**

**50-60 min, 21-25% B**

**60-70 min, 25-30% B**

**70-85 min, 30-90% B**

**85-117 min, 90-2% B**

**117-118 min, 2% B**

**LC pump (vendor and model)? Agilent 1200**

**Column washing step? No**

**Data acquisition start in the gradient? 2 min into the gradient**

# Protein and peptide identification and characterization software

1. General features

1.1 Global descriptors

– Date stamp (as YYYY-MM-DD) **n/a**

– Responsible person (or institutional role if more appropriate); provide name, affiliation and stable contact information **Peter Barr-Gillespie, Oregon Hearing Research Center, gillespp@ohsu.edu, 503-494-2936**

– Software name, version and manufacturer **MaxQuant 1.5.1.2**

– Customizations made to that software **n/a**

– Availability of that software **maxquant.org**

– Location of the files generated; parameter files, spectral data (input/output) **n/a**

2. Input data and parameters

2.1 Input data

– Description and type of MS data **Thermo .RAW**

– Availability of MS data (source of data, file format) **ProteomeXchange; .RAW files**

2.2 Input parameters

– Databases queried; description and versions (including number of entries searched) **Mus_musculus_GRCm38_71_pep_all_add_GM1322_XIRP2 (Ensembl mouse 71 with added XIRP2 and GM1322 protein sequences)**

**50,883 entries searched**

– Taxonomical restrictions applied **n/a**

– Description of tool and scoring scheme **MaxQuant 1.5.1.2**

– Specified cleavage agent(s) **Trypsin/P**

– Allowed number of missed cleavages **2**

– Additional parameters related to cleavage **n/a**

– Permissible amino acids modifications **Acetyl (protein N-term); oxidation (M)**

– Precursor-ion and fragment ion mass tolerance for tandem MS (when applicable) **n/a**

– Mass tolerance for PMF (when applicable) **n/a**

– Thresholds; minimum scores for peptides, proteins (probabilities, number of hits, other metrics) **n/a**

– Any other relevant parameters **n/a**

3. The output from the procedure

*The procedure might generate all or part of the elements described below (identified proteins, identified peptides, quantization information). Select the elements that apply.*

3.1 For identified proteins

– Accession code in the queried database **See data files**

– Protein description **See data files**

– Protein scores **See data files**

– Validation status **See data files**

– Number of different peptide sequences (without considering modifications) assigned to the protein **See data files**

– Percent peptide coverage of protein **See data files**

– Identity of supporting peptides **See data files**

– In the case of PMF, number of matched/unmatched peaks **n/a**

3.2 For identified peptides

– Sequence (indicate any deviation from the expected protein cleavage specificity) **See data files**

– Peptide scores **See data files**

– Chemical modifications (artefactual) and post-translational modifications (naturally occurring); sequence polymorphisms with experimental evidence (particularly for isobaric modifications) **See data files**

– Corresponding spectrum locus **n/a**

– Charge assumed for identification and a measurement of peptide mass error **See data files**

– Other additional information, when used for evaluation of confidence **See data files**

3.3 Quantitation for selected ions

– Quantitation approach (*e.g.* 4plex-iTRAQ, ICAT, cICAT, COFRADIC) **Relative iBAQ**

– Quantity measurement (*e.g.* integration of signals, use of signal intensity) **Integration of MS1 peak area**

– Data transformation and normalization technique (description of method and software) **MaxQuant 1.5.1.2 and Excel; see below**

– Number of replicates (biological and technical) **Four biological replicates per group**

– Acceptance criteria (including measure of errors) **n/a**

– Estimates of uncertainty and the methods for the error analysis, including the treatment of relevant systematic error effects and the treatment of random error issues **n/a**

– Results from controls (when described) **n/a**

4. Interpretation and validation

– Assessment and confidence given to the identification and quantitation (description of methods, thresholds, values, etc,) **n/a**

– Results of statistical analysis or determination of false positive rate in case of large scale experiments **n/a**

– Inclusion/exclusion of the output of the software are provided (description of what part of the output has been kept, what part has been rejected) **All contaminants rejected; all reversed entries are present in output spreadsheet but are not used in quantitation calculations**

5. MaxQuant group-specific parameters

Type-- Standard

Multiplicity-- 1

Labels-- None

Variable modifications-- Acetyl (Protein N-term); Oxidation (M)

Digestion mode-- Specific

Enzyme-- Trypsin/P

Max. missed cleavages-- 2

Match type-- Match from and to

Instrument type-- Orbitrap

First search peptide tolerance-- 20 ppm

Main search peptide tolerance-- 4.5 ppm

Individual peptide mass tolerance-- Yes

Isotope match tolerance-- 2 ppm

Centroid match tolerance-- 7.5 ppm

Time valley factor-- 1.4

Isotope time correlation-- 0.6

Theoretical isotope correlation-- 0.6

Recalibration unit-- ppm

Use MS1 centroids-- No

Use MS2 centroids-- No

Intensity dependent calibration-- No

Min. peak length-- 2

Max. charge-- 7

Min. score for recalibration-- 70

Advanced peak splitting-- No

Intensity threshold-- 500

Intensity determination-- Value at maximum

Label-free quantitation-- None

Max. number of modifications per peptide-- 5

Min. time-- NaN

Max. time-- NaN

Cut peaks-- Yes

Additional var mods for special proteins-- No

Separate variable modifications for first search-- No

6. MaxQuant global parameters

Global parameters

Fixed mods-- carbamidomethyl (C)

Re-quantify-- no

Match between runs-- no

Decoy mode-- Revert

Special AAs-- KR

Include contaminants-- Yes

Separate FASTA file for first search-- No

PSM FDR-- 0.01

Protein FDR-- 0.01

Min. peptide length-- 7

Min. razor + unique peptides-- 1

Min. unique peptides-- 0

Min. score for modified peptides-- 40

Min. delta score for unmodified peptides-- 0

Min. delta score for modified peptides-- 6

Base FDR calculations on delta score-- No

Razor protein FDR-- Yes

Split protein groups by taxonomy ID-- No

Filter labeled amino acids-- Yes

Second peptides-- Yes

Dependent peptides-- No

Min ratio count-- 2

Peptides for quantitation--Unique + razor

Modifications used in protein quantitation-- Acetyl (Protein N-term); Oxidation (M)

Discard unmodified counterpart peptide-- Yes

Separate LFQ in parameter groups-- No

Stabilize large LFQ ratios-- Yes

iBAQ-- Yes

Advanced site intensities-- Yes

# Peptide and protein quantification analysis

***1. General features***

– Experiment identifier or name **n/a**

– Responsible person or role **Peter Barr-Gillespie, Oregon Hearing Research Center, gillespp@ohsu.edu, 503-494-2936**

– Quantitative approach **Relative iBAQ; label-free extracted ion chromatograms, with intensity converted to iBAQ and iBAQ converted to riBAQ (Krey et al., J. Proteome Res. 13, 1034; 2013).**

***2. Experimental design and sample description***

2.1. Experimental design

– Groups **P4-P6 ("P5") mouse hair bundles; P5 mouse utricles; P21-P25 ("P23") mouse hair bundles; P23 mouse utricles**

– Biological and technical replicates **4 biological replicates for each group**

2.2. Sample / Assay description

– Labeling protocol (if applicable) **n/a**

– Sample description **n/a**

Sample name **n/a**

Sample amount **Hair bundles derived from 100 utricles or 10 whole utricles**

Sample labeling with assay definition, i.e. MS run / data set together with reporting ion mass, reagent or isotope labeled amino acid **n/a**

Replicates and/or groups **Six gel slices per biological replicate**

Isotopic correction coefficients **n/a**

Internal references **n/a**

***3. Input data***

*Description and reference of the dataset used for quantitative analysis (no actual values).*

– Input data type **Thermo .RAW**

– Input data format **n/a**

– Input data merging **Gel slice data for given replicate merged**

– Availability of the input data **ProteomeXchange**

***4. Protocol***

*Description of the software and methods applied in the quantitative analysis (including transformation functions, aggregation functions and statistical calculations).*

4.1. Quantification software name, version and manufacturer **iBAQ calculated with MaxQuant 1.5.1.2; all other calculations done with Microsoft Excel**

4.2. Description of the selection and/or matching method of features, together with the description of the method of the primary extracted quantification values determination for each feature and/or peptide **By MaxQuant**

4.3. Confidence filter of features or peptides prior to quantification **By MaxQuant**

4.4. Description of data calculation and transformation methods

– Missing values imputation and outliers removal **n/a**

– Quantification values calculation and / or ratio determination from the primary extracted quantification values **riBAQ for each protein or protein group is iBAQ divided by the sum of all non-contaminant, non-reversed iBAQ values**

– Replicate aggregation **Replicates averaged together**

– Normalization **riBAQ intrinsically normalizes**

– Inference protocol for calculating protein quantification values from peptide quantification values **By MaxQuant**

– Protocol specific corrections **n/a**

4.5. Description of methods for (statistical) estimation of correctness **Reversed database used for FDR estimate at protein level**

4.6. Calibration curves of standards **Assumption is that riBAQ is proportional to relative molar abundance (Krey et al., J. Proteome Res. 13, 1034; 2013)**

***5. Resulting data***

*The actual quantification values resulting from your quantification software together with their estimated confidence*

*5.1.* Quantification values at feature and/or at peptide level

– Primary extracted quantification values for each feature, with their statistical estimation of correctness **n/a**

– Quantification values for each peptide as a result of the aggregation of the values of the previous section (5.1.1), with their statistical estimation of correctness **Reversed database used for FDR estimate of 1% at peptide level**

5.2. Quantification values at protein level

– Basic / raw quantification values with statistical estimation of correctness **Reversed database used for FDR estimate of 1% at protein level**

– Transformed / aggregated / combined quantification values of the proteins at group level, with their statistical estimation of correctness **FDR re-evaluated after protein grouping**
